# The effects of AMPA receptor blockade on resting magnetoencephalography recordings

**DOI:** 10.1177/0269881117736915

**Published:** 2017-10-31

**Authors:** Bethany C Routley, Krish D Singh, Khalid Hamandi, Suresh D Muthukumaraswamy

**Affiliations:** 1 Cardiff University Brain Research Imaging Centre (CUBRIC), Cardiff University, Cardiff, UK; 2 The Epilepsy Unit, University Hospital of Wales, Cardiff, UK; 3 School of Pharmacy, The University of Auckland, Auckland, New Zealand; 4 School of Psychology, The University of Auckland, Auckland, New Zealand

**Keywords:** Magnetoencephalography, glutamate, α-amino-3-hydroxy-5-methyl-4-isoxazolepropionic acid, perampanel

## Abstract

The ionotropic N-methyl-D-aspartate and α-amino-3-hydroxy-5-methyl-4-isoxazolepropionic acid (AMPA) receptors of the glutamatergic neurotransmitter system are of fundamental importance to healthy brain function. Neuroimaging studies in humans have previously been conducted using various drugs that interact with N-methyl-D-aspartate glutamate receptors, but no such studies have investigated AMPA receptor signalling. The recent approval of perampanel (Fycompa) for use in humans provides a means to specifically study the role of AMPA receptors in the pharmacological basis of neuroimaging signals. Twenty male subjects participated in this placebo-controlled crossover study that consisted of two study days separated by a minimum two-week washout period. On one occasion participants ingested a 6 mg dose of perampanel, and on the other a placebo. Ten minutes of wakeful rest was recorded before and after each dose using magnetoencephalography. Subjective ratings of intoxication were significantly higher following drug than placebo. Cluster-based randomisation testing of sensor-level magnetoencephalography data showed significant drug-induced increases in low frequency power (1–4 Hz, 4–8 Hz, 8–13 Hz, 13–30 Hz), along with a significant decrease in the high gamma range (50–90 Hz). We also observed selective increases in functional connectivity in the alpha and beta bands. The findings are consistent with preclinical work and are similar to the spectral profile of other anti-epileptic drugs.

## Introduction

The magnetoencephalography (MEG) signal arises from synchronous activity within neuronal assemblies, in particular the summed post-synaptic potentials of cortical pyramidal cells (Lopes de Silva, 2010). By logical extension, MEG data are influenced by a complex interplay of neurochemical systems. Activity across the frequency range typically measured using MEG (0≥100 Hz) can be selectively modified by pharmacological agents acting on neurotransmitters including γ-aminobutyric acid (GABA; Hall et al., 2010; Saxena et al., 2013) serotonin (Muthukumaraswamy et al., 2013) and dopamine (Moran et al., 2011), among others. Further, this pharmaco-MEG approach can be applied to the study of varied cognitive processes (Hall et al., 2011; Linssen et al., 2014), and a number of neuropsychological diseases and neuropsychiatric disorders (Bajo et al., 2015; Franzen et al., 2013; Heinrichs-Graham et al., 2014; Wilson et al., 2013). Whereas GABA is the primary inhibitory neurotransmitter in the human brain, glutamate is the primary excitatory neurotransmitter. Ionotropic glutamate receptors, particularly N-methyl-D-aspartate (NMDA) and α-amino-3-hydroxy-5-methyl-4-isoxazolepropionic acid (AMPA) subtypes, are of fundamental importance in neuronal signalling. Both AMPA and NMDA receptors co-exist on most excitatory synapses within the central nervous system, but crucially possess differing kinetic properties that coupled together define the time course of synaptic transmission (Traynelis et al., 2010). Activation of the AMPA receptor is a rapid process whereby channels allowing influx of sodium ions open and close within 2–3 ms (Dingledine et al., 1999). NMDA receptors, however, are more permeable to calcium ions and possess a slower rise time (~20 ms), with a several-hundred-millisecond delay in closing (Dingledine et al., 1999). Voltage-dependent regulation of the glutamate receptors by endogenous ions not only defines the time course of synaptic transmission but is also thought to be key in synaptic plasticity (Traynelis et al., 2010). Furthermore, dysfunction in glutamatergic systems is implicated in many neurological and neuropsychiatric disorders including schizophrenia (Goff and Coyle, 2014; Olney and Farber, 1995), mood disorder (Sanacora et al., 2012) and Alzheimer’s disease (Hynd et al., 2004).

There have been various neuroimaging studies of compounds acting on the GABAergic system using both functional magnetic resonance imaging (fMRI) (Licata et al., 2011) and electroencephalography (EEG)/MEG (Ahveninen et al., 2007; Lozano-Soldevilla et al., 2014; Saxena et al., 2013). Fewer studies have investigated the glutamate system and of those, most have focused on agents which interact with NMDA receptor (Downey et al., 2016; Korostenskaja et al., 2007; Northoff et al., 2005; Sanacora et al., 2014; Shaw et al., 2015). But there are, to our knowledge, no neuroimaging studies to-date that have used a selective AMPA receptor drug. Until recently, such a compound was not available for use in humans. Perampanel is a new anti-epileptic drug (approved 2012) that acts as a non-competitive antagonist of the AMPA receptors, and so reduces the actions of glutamate at the synapse. The compound is highly-selective, and at therapeutic doses displays no affinity to the other ionotropic glutamate receptors (NMDA or kainate; Rogawski and Hanada, 2013). This drug has been licensed in the USA and EU since 2012 as an adjunctive medication in the treatment of refractory partial-onset epileptic seizures.

Conducting non-clinical intervention studies with pharmaco-MEG not only enables study of the actions of pharmacological agents non-invasively in humans, but also helps to develop understanding of the neurotransmission dynamics underlying the MEG signal (Muthukumaraswamy, 2014). Using a task-free paradigm enables us to investigate the effects of different compounds across a variety of frequency bands within one recording. Previous animal studies suggest that both low and high frequency oscillations are affected by AMPA receptor activity. For example, an EEG study in conscious rats demonstrated dose-dependent increases in power across the 1–30 Hz spectrum following administration of two AMPA antagonists (Sebban et al., 2002). At higher frequencies, Oke et al. (2010) showed that application of the AMPA antagonist SYM-2206 to slice preparations taken from rat visual cortex almost abolished both low (~50 Hz) and high (~80 Hz) gamma oscillations. Furthermore, concurrent increases in low frequency oscillatory power (1–4 Hz and 7–13 Hz) and decreases in high frequency power (30–60 Hz) have been found in a task-driven study following administration of AMPA antagonists to the visual cortex of monkeys in vivo (Herrero et al., 2013).

Therefore, the present study aimed to investigate the actions of glutamate in the human brain, by utilising the interaction between perampanel and AMPA receptors at the synapse. Here, we describe the oscillatory profile of perampanel during task-free MEG recordings in a group of healthy volunteers. Further, we reconstruct source locations of drug-effects and investigate drug-related changes to broadband functional connectivity. Based on previous in-vivo (Herrero et al., 2013; Sebban et al., 2002) and in-vitro work (Oke et al., 2010), we expected to see an increase in power in low frequency bands, coupled with a decrease in power of gamma band activity.

## Methods and materials

### Participants

Twenty healthy volunteers (mean age 22.9 years, standard deviation (SD) 3.75; mean weight 75.6 kg, SD 8.2) participated in the study. Inclusion criteria were that participants be males between 18–45 years old, non-smokers, with a body mass index of 18–30 kg/m². We restricted participation to males only due to prior instances of adverse events in pharmaco-MEG studies with female participants (Hamandi et al., 2014) and to avoid potentially confounding effects of the menstrual cycle, which is known to affect resting EEG (Creutzfeldt et al., 1976; Solis-Ortiz et al., 1994). Exclusion criteria included personal history of neuropsychiatric or neurological disorder, current recreational or prescription drug use, ongoing health problems (including liver and cardiovascular function) and contraindications for MEG/magnetic resonance imaging (MRI). Participants were additionally screened for alcohol misuse with the Alcohol Use Disorders Identification Test (AUDIT; Saunders et al., 1993); all participants scored below the threshold for alcohol dependence (≥16; mean score 7.1, SD 3.9). Participants were required to abstain from alcohol for 72 h prior to study sessions, and from use of illicit substances and ‘legal highs’ for seven days prior. All procedures were approved by the UK National Research Ethics Service (South East Wales), and a description of task-related MEG activity from the same recording days is available elsewhere (Muthukumaraswamy et al., 2016).

### Design and procedure

Participants were scanned on two separate days in a single-blind, placebo-controlled crossover design. Study sessions were separated by a minimum period of 14 days to allow for drug washout. Each session took place at approximately the same time of day, and session order (drug/placebo) was counterbalanced across participants. During each study session a ‘pre-dose’ MEG recording was obtained, following which participants orally ingested a capsule containing either 6 mg of perampanel (Fycompa) or an unmarked vitamin E placebo. A further ‘post-dose’ MEG recording was obtained two hours after ingestion, at which time perampanel is expected to have reached peak plasma level (Templeton, 2010). As part of each MEG scan a 10-minute resting recording was obtained, during which time participants were instructed to remain relaxed but alert with their eyes open and fixated on a red circle presented at the centre of the screen. The fixation point was displayed on a Sanyo PLC-XP41 projector with a screen resolution of 1024×768 and refresh rate of 60 Hz. All recordings were made with participants lying supine in the scanner. Just prior to each MEG recording, participants completed a battery of psychological questionnaires including the Subjective High Assessment Scale (SHAS; Schuckit, 1980) and Biphasic Alcohol Effects Scale (BAES; Martin et al., 1993) to measure subjective drug experience, and the State Hostility Scale (SHS; Anderson et al., 1995) to quantify experience of potential side effects.

### MEG recordings

Whole-head MEG recordings were made using a CTF-Omega 275 channel system, sampled at 1200 Hz and analysed in third-order gradiometer mode. Four of the 275 channels were turned off due to excessive sensor noise. An additional 29 reference channels were recorded for noise cancellation purposes. Eye movements and blinks were monitored using vertical and horizontal electrooculogram (EOG) recordings, and an electrocardiogram (ECG) was also recorded.

For source localisation, a 1 mm isotropic fast spoiled gradient echo (FSPGR) anatomical MRI scan was obtained, either on a different day to the MEG study days or available from previous study participation at the Cardiff University Brain Research Imaging Centre (CUBRIC). To achieve MEG/MRI co-registration, electromagnetic coils were placed at fixed distances from anatomical landmarks (10 mm anterior to left and right tragus, 10 mm superior to nasion) and localised immediately before and after each recording. Fiduciary locations were later manually marked on the anatomical MRI.

### Sensor level analysis

Offline, the data were downsampled to 600 Hz. A band-pass filter of 1–150 Hz was applied to the continuous data in order to minimise edge effects, and the data was then epoched into two-second segments. Each epoch was visually inspected and those containing gross muscle artifacts (e.g. jaw clenches) were removed from subsequent analysis. To complete pre-processing we applied independent component analysis to the data and rejected artifacts including eye movements and cardiac noise, based on topography and waveform patterns of each component. For the following analysis, the frequency bands used were: delta (1–4 Hz), theta (4–8 Hz), alpha (8–13 Hz), beta (13–30 Hz), low gamma (30–50 Hz) and high gamma (50–100 Hz). These bandings are consistent with similar previous work (Nutt et al., 2015).

We examined the power spectra of various frequency bands in sensor space, using the FieldTrip toolbox (Oostenveld et al., 2011). The analysis pipeline is similar to that described previously (Nutt et al., 2015) and uses a dose subtraction approach to probe drug-induced changes. The pre-processed data were first converted to planar gradient formation and frequency analysis was conducted using Hanning-windowed fast Fourier transforms. The gradients over both planar directions were then combined to obtain a single positive-valued number under each sensor. In this sensor configuration, sources can be assumed to lie directly underneath local maxima on field maps, thus allowing the results of this analysis to be more easily interpretable (Bastiaansen and Knosche, 2000). Difference images were then created by subtracting the pre-dose spectra from the post-dose spectra for each condition (drug/placebo) and participant, according to the frequency bands defined above. Statistical differences between the drug and placebo conditions were determined used Monte-Carlo permutation testing of *t*-statistics on these difference images (5000 permutations, cluster-based multiple comparisons correction applied).

Visual inspection of the power spectra indicated that there may be a drug-related slowing of alpha oscillations. So, in addition to changes in oscillatory power we also chose to selectively examine the effects of perampanel on alpha frequency. We used an approach to peak frequency estimation with quality control that has previously been described for gamma oscillations (Magazzini et al., 2016). Here, the single trial spectra were averaged separately for each condition and time-point (pre-placebo, post-placebo, pre-drug and post-drug) and the channel with greatest alpha power in each case selected. Single-trial spectra in the peak channel were then resampled (with replacement) using 10,000 iterations of bootstrapping, re-averaged, and peak alpha frequency for each participant was defined as the bootstrapped mode in the 8–13 Hz range. To control for data quality, we used the distribution of peak frequency estimations generated by the bootstrapping, and included data for any participant only when at least 50% of bootstrap iterations occurred within a frequency window of ±1Hz around the bootstrapped mode, for all four conditions. No participants were excluded under this criterion. Differences in peak frequency were then analysed using a 2×2 repeated measures analysis of variance (ANOVA), using factors drug (placebo and perampanel) and time (pre-dose and post-dose).

### Source level analysis

We additionally investigated drug-related changes in source space power and connectivity patterns. For these analyses we used the preprocessed data previously described and applied a series of linearly constrained minimum variance (LCMV) beamformers implemented in Fieldtrip (Oostenveld et al., 2011) to reconstruct sources on a 6 mm grid using covariance matrices derived from six frequency bandpass filtered versions of the datasets: 1–4 Hz, 4–8 Hz, 8–13 Hz, 13–30 Hz, 30–50 Hz and 50–90 Hz. For each band, the beamformer weights were normalised using a vector norm (Hillebrand et al., 2012). The band limits here are consistent with the sensor-space analysis previously described, and with previous work investigating resting state MEG (Brookes et al., 2011; Hall et al., 2013; Muthukumaraswamy et al., 2013). The data were normalised to the Montreal Neurological Institute (MNI) template, and reduced to 90 nodes according to the automatic anatomical labelling (AAL) atlas (Hillebrand et al., 2012). We opted for a single representative in each of the 90 regions of interest (ROIs) by automatically selecting the virtual sensor with the greatest percentage change in power over the recording.

For the connectivity analysis, we used an amplitude coupling approach that assesses temporal interactions between the amplitude envelopes of brain sources. In such an approach, stronger correlations between the time series of two regions is taken as indication of stronger functional connectivity between those areas. We used the 90 virtual sensor time series in an atlas-based approach to connectivity estimation (Hillebrand et al., 2012). In order to correct for source leakage and minimise the effect of spurious correlations, we applied a symmetric orthogonalisation procedure (Colclough et al., 2015). This two step approach first finds the set of orthonormal vectors that are closest to the original set of ROI time series. Then, the magnitude and orientation of these vectors is iteratively adjusted to reach a corrected solution that varies minimally from the original uncorrected time series. Ultimately, this produces a mutually orthogonal set of time series for the included ROIs where zero-lag correlations have been removed and remaining correlations are assumed to reflect true biological coupling (Colclough et al., 2015). A Hilbert transform is computed on these orthogonalised time series and the amplitude envelope extracted. The orthogonalised envelopes are despiked and downsampled to 1Hz. We then cross-correlate all 90 envelopes to derive a connectivity matrix based on amplitude coupling across regions. Correlation coefficients within this matrix, for each dataset, are then transformed to a normalised *z*-scores using Fisher’s transform and an estimate of the effective degrees of freedom from the raw time series. These normalised *z*-scores are then suitable for taking forward for statistical analyses. We sum along the rows of this *z* matrix to derive a measure of ‘connection strength’, representing in a single metric how connected each region is to every other region. As in the sensor space analysis, we create difference scores for each participant by subtracting the pre-dose connection strength measures from the post-dose measures for both perampanel and placebo. We then conduct a randomisation test (10,000 permutations) on the difference between these vectors, with omnibus correction for multiple comparisons (Nichols and Holmes, 2002).

In order to generate a candidate source distribution for drug-related power changes, we projected raw power values through the previously generated beamformer weights to derive source power in each of the 90 AAL regions, for each participant. To test for statistical significance, we followed the same steps as the prior analysis and first subtracted the pre-dose from the post-dose source power values for both perampanel and placebo in each frequency band. We then used randomisation testing on the differences to determine areas of significant drug-related power change.

## Results

### Subjective experience of drug

The behavioural measures indicate changes to subjective experience following dosage of the drug, particularly increased feelings of drowsiness or sedation. Descriptive statistics for all scales can be found in the Supplementary Material, along with group averages for the individual scale items on the BAES and SHAS. In the BAES, there was an average 13.5 point increase on the sedative subscale following perampanel dosage (compared to 0.6 point increase on placebo), with the greatest increase seen on the ‘sedated’ item (2.75 point increase, maximum score per item is five points). Similarly, in the SHAS, many items increased by an average of 10 points or more following drug dose (maximum score per item is 100), with the greatest increase reported in feelings of sleepiness and concentration (31.1 and 24.8 point average change, respectively). As well as somnolence, perampanel has significant association with adverse events of dizziness and irritability (Zaccara et al., 2013). Reports of dizziness on the SHAS were increased by 18.9 points following perampanel administration in this study, but there was no increase on the ‘irritable’ item of the SHS.

**Figure 1.**
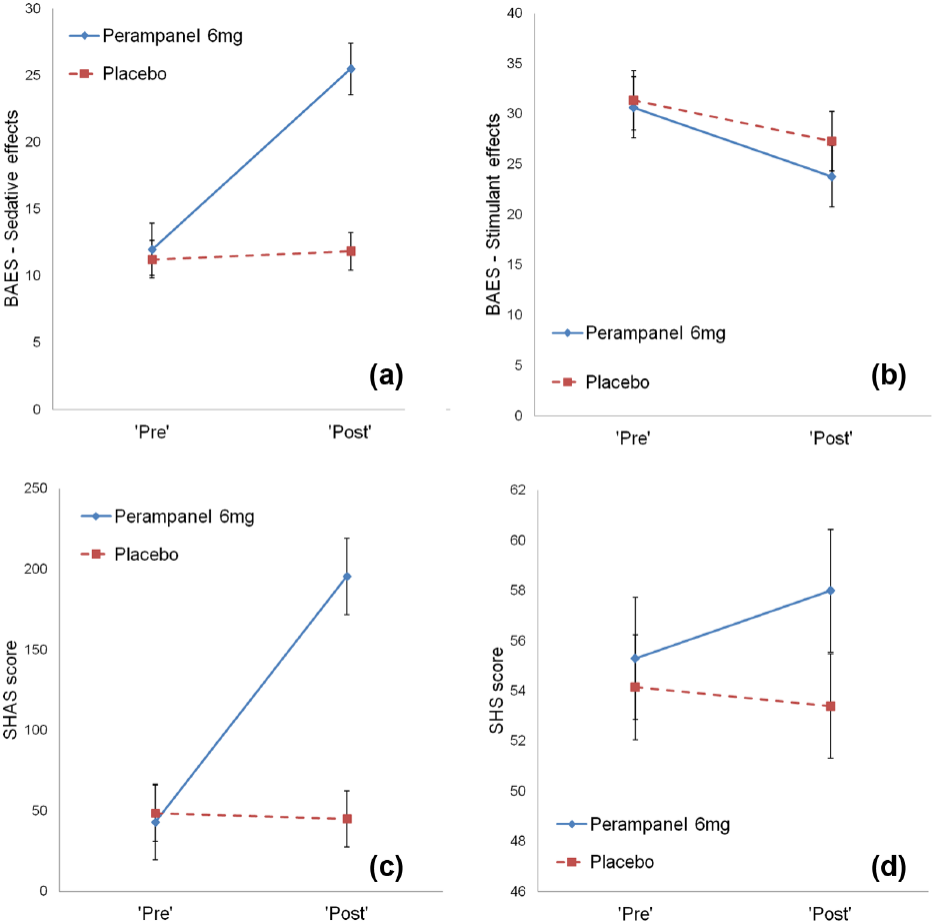
Differences in mean scores on psychometric scales between ‘pre’ and ‘post’ time points for drug and placebo, (a) and (b) for the Biphasic Alcohol Effects Scale (BAES), (c) Subjective High Assessment Scale (SHAS) and (d) State Hostility Scale (SHS). *Indicates significant interaction terms (*p*<0.01).

A series of 2×2 ANOVAS revealed significant drug-time interactions in both the sedative scale of the BAES (Figure 1(a); *F*(1,19)=28.4, *p*<0.01), and the SHAS (Figure 1(c); *F*(1,19)=23.7, *p*<0.01). There were no significant interaction effects in the stimulant scale of the BAES (Figure 1(b); *F*(1,19)=0.8, *p*=0.37), or the SHS (Figure 1(d); *F*(1,19)=2.4, *p*=0.14). All participants were able to correctly identify the session order following completion of the study.

### Power and frequency changes in sensor space

Following artefact rejection from the original trials, there were comparable trial numbers left in each condition: placebo pre-dose=293 (SD=8.1), placebo post-dose=292 (SD=6.4), drug pre-dose=294 (SD=4.5), drug post-dose=293 (SD=8.9) indicating preserved data quality following drug administration.

**Figure 2.**
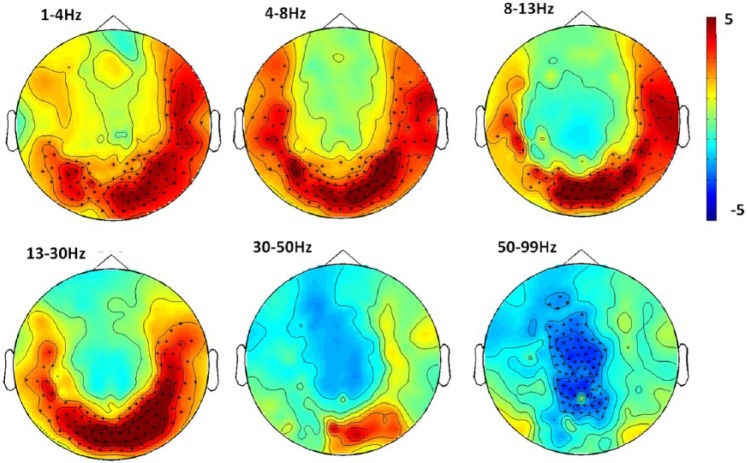
Contrast performed on drug-placebo difference spectra, in the frequency bands: δ, θ, α, β, low γ, high γ. Red indicates a relative increase in power following drug and blue indicates relative decrease. Units are *t* statistics and * indicates significant sensor clusters (*p*<0.01, corrected for multiple comparisons).

Drug-induced changes in power were observed in almost all frequency bands. In the lower bands (δ–β) we found a significant increase (*p*<0.01) in power focused around posterior sensors. There were no significant changes to power in the low gamma range, but a significant decrease in power (*p*<0.01) over central parietal sensors in the high gamma range. Figure 2 topographically shows the drug-related effects on the power spectrum in each frequency band.

**Figure 3.**
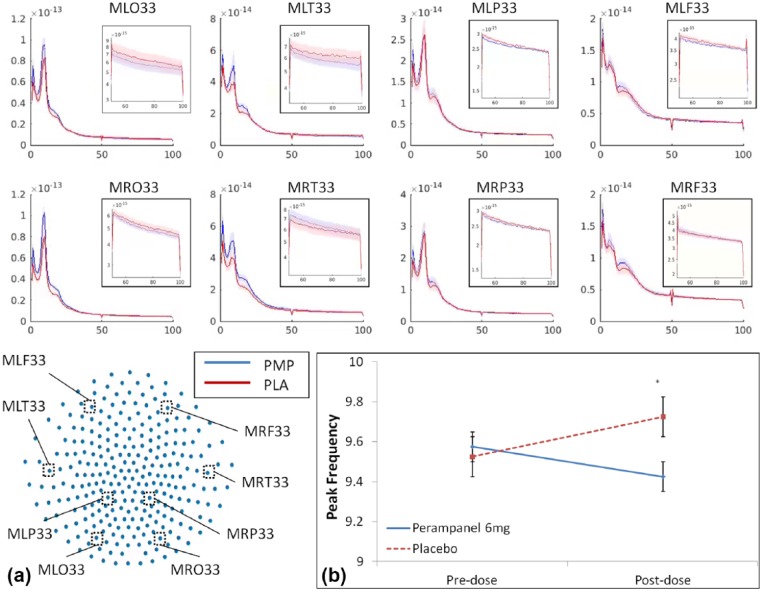
(a) Power-frequency plots for post-dose conditions at single sensors in the occipital, temporal, parietal and frontal regions. Frequency is plotted on the *x*-axis and power on the *y*-axis. Sensor locations are shown in the bottom-left schematic. Shaded bars are standard errors. Inset plots show 50–100 Hz on a log-scale. (b) Changes to peak frequency in the alpha (8–13 Hz) band, for pre-dose and post-dose drug and placebo conditions. Bars show standard error. *Indicates significant interaction. PMP: perampanel; PLA: placebo.

The broadband effects of perampanel on oscillatory power at single sensor locations around the head are shown in Figure 3(a). Inspection of these plots indicates that in addition to increasing power at low frequencies and decreasing power at higher frequencies, perampanel may also cause frequency slowing in the alpha band. Subsequent analysis of peak frequency using the bootstrapping with quality control method detailed above confirmed this, with the drug×time interaction term of a 2×2 repeated measures ANOVA showing a relative decrease in frequency following the 6 mg dose of perampanel (Figure 3(b); *F*(1,19)=5.444, *p*=0.03).

### Power and connectivity changes in source space

**Figure 4.**
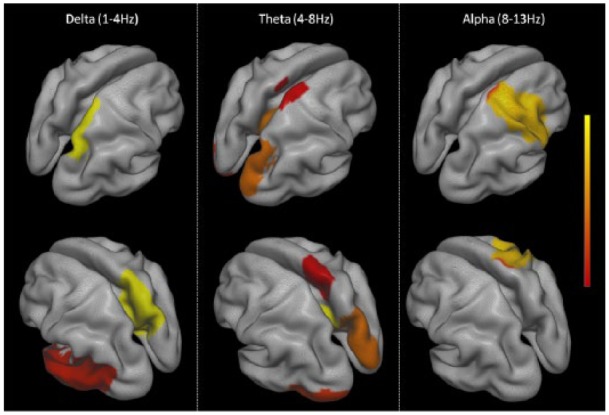
Areas of significant drug-induced power increase (delta-alpha frequencies). Source localised power was compared at a single virtual sensor for each of the 90 automatic anatomical labeling (AAL) regions studied using randomisation testing, and only those regions that show significant (*p*<0.05) drug induced-changes following correction for multiple comparisons are displayed here.

Following source estimation, we identified a candidate source distribution for drug-induced power changes using the power values for single virtual sensors in 90 AAL regions. Significant increases in source power were observed in low frequencies (delta-alpha) following perampanel compared to placebo (Figure 4). In the delta band, significantly increased power was observed in the right precuneus and left middle temporal gyrus. For the theta band, there were widespread statistically significant drug-related power increases (right olfactory cortex, left posterior cingulate gyrus, right posterior cingulate gyrus, right cuneus, left inferior occipital cortex, right paracentral lobule, left Heschl gyrus). In the alpha band, drug-induced power increases were significant in the right precentral gyrus and right paracentral lobule. There were no statistically significant drug-related changes of source power in the beta or gamma bands. This pattern of source localised power changes is somewhat consistent with power changes in the sensor space analysis but effects are less pronounced.

**Figure 5.**
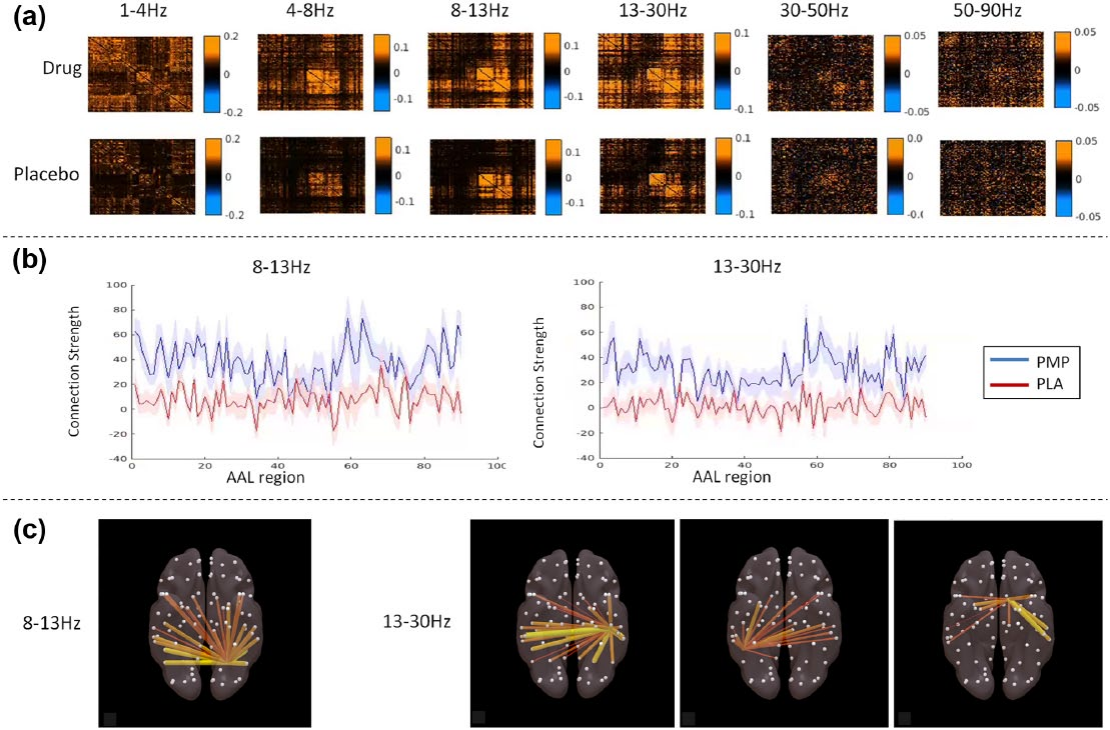
(a) Post-dose connectivity matrices for each of the frequency bands studied. Each point in the plot indicates the correlation of one automatic anatomical labeling (AAL) region with another (90×90). (b) For alpha (8–13 Hz; top) and beta (13–30 Hz; bottom), difference scores (post dose - pre dose) for *z*-corrected mean connection strength for all AAL regions. (c) Connectivity plots for AAL regions that exhibit significant drug-induced changes in connection strength, in the alpha band (left superior parietal lobule) and beta band (left postcentral gyrus, right inferior parietal gyrus and left caudate). Grey circles indicate AAL nodes. Increased coupling between nodes is plot on red-yellow scale and stronger connections have greater line thickness. PMP: perampanel; PLA: placebo.

We also assessed drug-related changes to connectivity in each of the frequency bands. The average adjacency matrices (90×90 regions) for each frequency band are shown in Figure 5(a). In both the drug and placebo conditions, the connectivity takes on most structure in the alpha beta ranges, with a ‘hub’ region of increased connectivity in occipital and parietal areas. Stronger colouration in the post-drug matrices indicates that amplitude coupling is increased across the low frequency ranges following perampanel dose. However, the only connectivity increases that survive statistical testing with multiple comparisons correction can be seen in the alpha and beta bands (Figure 5(b)). In both cases, these changes localise mainly to parietal regions. For the alpha band, significantly increased connectivity is observed following drug dose in the left superior parietal lobule (area 59, *p*=0.04). For the beta band, this increased connectivity is observed in the left postcentral gyrus (area 57, *p*<0.01), right inferior parietal gyrus (area 62, *p*<0.01) and left caudate (area 71, *p*=0.04). The regions that are most strongly coupled to these significant areas are shown in Figure 5(c).

**Figure 6.**
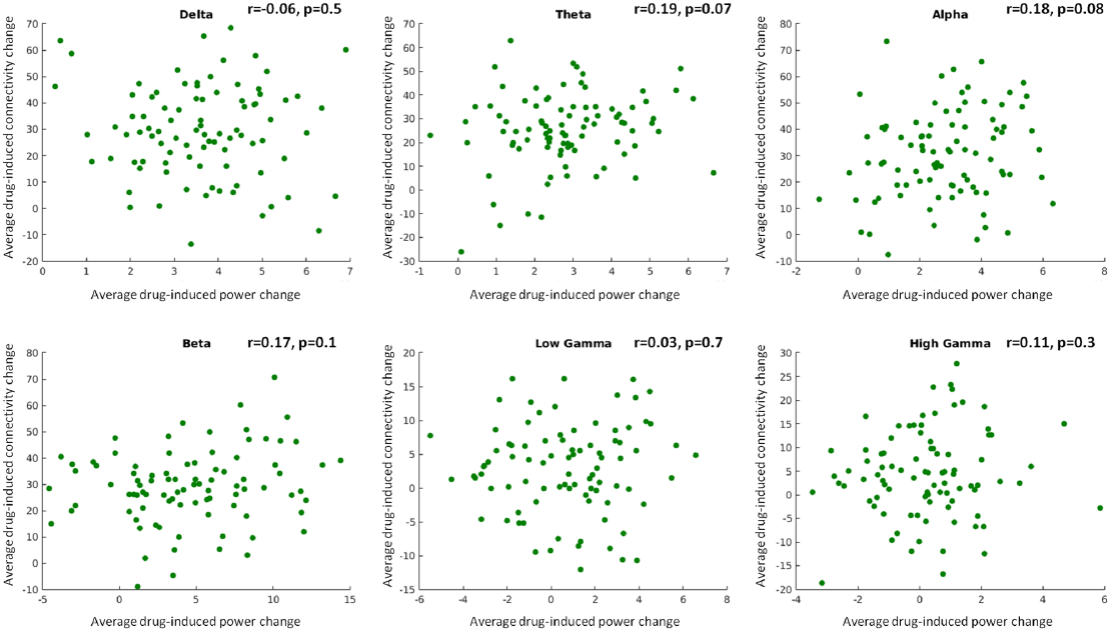
Scatter plots of the mean effects of drug administration connection strength and power in each frequency band (perampanel – placebo). Each point plotted represents the drug-induced change to power and connectivity for one automatic anatomical labeling (AAL) region, averaged across participants. Correlations between the mean effects were not statistically significant in any frequency band. Units are arbitrary due to the beamformer weights normalisation process that is needed to correct for biases introduced by non-uniform sensor-noise projection (Hillebrand et al., 2012).

In order to ensure that drug-related changes to power could not account for drug-related changes to connectivity, we correlated mean drug-effects on source power with mean drug-effects on connection strength. For each participant, we subtracted pre-dose from post-dose values of source power and connection strength. We then subtracted the placebo dose-difference values from perampanel dose-difference values and averaged across participants to derive a single value of drug-related power and connectivity in each AAL region. These mean drug-effects for each frequency band are plot in Figure 6. There were no significant correlations between mean effects in source power and connection strength for any frequency band: delta (*r*=−0.06, *p*=0.5), theta (*r*=0.19, *p*=0.07), alpha (*r*=0.18, *p*=0.08), beta (*r*=0.17, *p*=0.1), low gamma (*r*=0.03, *p*=0.7) or high gamma (*r*=0.11, *p*=0.3).

In order to relate neural measures with measures of subjective wellbeing, we correlated the average power and connection strength for each participant at the post-perampanel time point with individual scores on the SHAS. There were no significant correlations between either measure in any frequency band. Power: Delta (*r*=0.09, *p*=0.7), theta (*r*=−0.05, *p*=0.8), alpha (*r*=−0.13, *p*=0.5), beta (*r*=−0.18, *p*=0.4), low gamma (*r*=−0.16, *p*=0.4), high gamma (*r*=−0.13, *p*=0.5). Connection strength: Delta (*r*=0.07, *p*=0.7), theta (*r*=0.18, *p*=0.4), alpha (*r*=−0.04, *p*=0.8), beta (*r*=−0.04, *p*=0.8), low gamma (*r*=−0.16, *p*=0.4), high gamma (*r*=−0.13, *p*=0.5).

## Discussion

We report here for the first time the impact of the AMPA antagonist perampanel on signal power across 1–100 Hz in resting-state EEG/MEG recordings in humans. We predicted that dosage of perampanel would increase MEG power in low frequency bands and decrease power in higher bands, and this hypothesis was supported. Following perampanel administration, we saw an increase in posterior power in lower frequency bands, but decreased power in the high gamma range over parietal sensors. Further, we also investigated the effect of perampanel on functional connectivity in source space. We found selective increases in functional connectivity following drug dose. Analysis of psychometric scales confirmed increased subjective ratings of intoxication following the dose of perampanel compared to placebo.

Participants reported feeling more intoxicated following perampanel dose compared to placebo, and on completion of the study all were able to correctly guess their session participation order. The significant effect of perampanel on the SHAS is consistent with these subjective reports, and findings of significant drug effects in the sedative but not stimulant scale of the BAES is consistent with the drug’s mechanism of action. In some cases perampanel can have psychiatric side effects including increased aggression and hostility (FDA, 2012). However, the results of the SHS suggest that there were no such side effects in the present study, likely due to only a single administration of the drug. Although the experiment was run under single-blind conditions the subjective effects participants’ experienced effectively unblinded them from the intervention. This is a common issue in many psychopharmacology experiments. That said, given the basic physiological measures reported here (as opposed to clinical responses), and the similar data quality between conditions, we would argue that the effective unblinding is unlikely to have affected our central results.

To the best of our knowledge this is the first human neuroimaging study of the effects of a selective AMPA compound and as such the results cannot be compared to previous human studies. However, our results are consistent with previous animal work showing suppression or near elimination of gamma oscillations and increase in low frequency oscillations following administration of AMPA antagonists (Herrero et al., 2013; Oke et al., 2010; Sebban et al., 2002). The reduction in gamma oscillations found in the sensor space analysis did not carry over into the source localised power effects, perhaps due to the low SNR of gamma frequency oscillations and additional processing required to move to source space. Nevertheless, the source localised power measures also show drug-related increases in low frequencies, consistent with animal work (Sebban et al., 2002).

It becomes more difficult to compare the connectivity analyses to previous literature. It is worthwhile to note that the regions showing increases in connectivity following perampanel dose are distinguishable from those which showed a straightforward power increase in the sensor analysis. Generally speaking, we found that connectivity was higher in parietal regions in all conditions. This is reflective of previously documented fMRI connectivity hubs in parietal regions, including the inferior parietal cortex and postcentral gyrus (Buckner et al., 2009; Tomasi and Volkow, 2011). There is some evidence that focal epilepsies are associated with reductions in connectivity in various networks, including those recruiting parietal regions (e.g. sensorimotor networks; Liao et al., 2010; Luo et al., 2012). So, the increase in connectivity following perampanel dose may relate to the seizure controlling mechanism of the drug.

We found no relationship between a subjective measure of wellbeing (SHAS) and MEG results, perhaps indicating that general feelings of sedation cannot account for drug-related changes to the MEG signal. However, subjective measures are problematic in that each participant has their own internal ‘yardstick’ which could make it difficult to tease out brain-psychometric relationships. Given that the glutamatergic system plays a key role in synaptic plasticity (McEntee and Crook, 1993) it is plausible to assume that manipulation of AMPA-R may affect learning and memory processes, though the precise role of this receptor in learning is still unclear (Riedel et al., 2003). Specific study of behaviours and cognitions related to altered glutamate levels were beyond the scope of the present work, so additional research is needed to disentangle these processes further.

Interestingly, NMDA antagonists (e.g. ketamine) seem to exhibit almost the opposite pattern of effects from the present study. Compounds of this type increase fast- and decrease slow background rhythms in both in-vivo mouse recordings (Lazarewicz et al., 2009) and human EEG (Hong et al., 2009). Furthermore, AMPA and NMDA signalling pathways have been shown to have differential effects on fMRI signals in rodents (Gsell et al., 2006). This suggests that it is too simplistic to consider generic glutamate effects on oscillatory activity, but rather the separation of specific receptors is critical to understanding the generation of EEG/MEG signals.

The similarities of the perampanel spectra to those of tiagabine reported by Nutt et al. (2015) is notable. Both drugs increase slow wave activity and decrease faster rhythms, thus shifting the brain to a less excitable state, but have marked differences in their mechanism of action. Whereas perampanel decreases the actions of glutamate at the synapse via allosteric blockade of AMPA receptors, tiagabine is a GABAergic drug that is thought to potentiate GABAergic inhibition by blocking reuptake (Meldrum, 1996). Both drugs are most commonly used as an adjunctive treatment for refractory partial seizures in epilepsy, so the similarities in spectral power changes may reflect the seizure-controlling mechanisms of both compounds. However, there are also differences apparent between the profiles of the two drugs, with effects of tiagabine being more spatially diffuse in the delta and theta bands and more frontally focused in alpha and beta bands, compared to the overall posterior pattern observed with perampanel.

We chose to complete the second MEG recording two hours after dosage, as a single dose <8 mg is expected to have reached maximum blood plasma level by this time (Templeton, 2010), but with a range from 30 min to 2 h, it is possible that we missed the peak for some participants. This being said, the combined effects of fast absorption rate and long terminal half-life of perampanel suggests that we would be unlikely to be more than 50 ng/mL from peak concentration for any given participant. Measuring plasma concentrations following dosage with the drug might have ensured the post-dose time points were collected at the optimum time. Additionally, collecting multiple post dose time points as has been done previously (Nutt et al., 2015) would give a more comprehensive picture of the full effects of the pharmokinetic profile on oscillatory activity, though the terminal half life range from 53–123 h means that collecting data over the full range would be difficult in practical terms. Furthermore, it would be interesting to study the oscillatory effects of perampanel at sustained doses, for example in epilepsy patients prior to commencement of perampanel and again at steady-state dose. However such studies in patient populations present practical difficulties through disease heterogeneity and variable concomitant medications.

In conclusion, the results reported here demonstrate that perampanel has widespread effects on MEG spectral power and functional connectivity at rest. This may be of interest when considering the seizure-controlling mechanism of the drug, particularly in view of the similarity with the tiagabine profile. Furthermore, the results taken with previous work on NMDA receptor antagonists highlight the sensitivity of MEG to specific receptor level changes within the glutamatergic system. Taken together, the findings indicate that MEG may be useful in determining the specific cortical effects of new and established drugs, and relating these to clinical or behavioural outcomes.

## Supplementary Material

Supplementary material

## References

[bibr1-0269881117736915] Ahveninen J Lin FH Kivisaari R et al (2007) MRI-constrained spectral imaging of benzodiazepine modulation of spontaneous neuromagnetic activity in human cortex. Neuroimage 35: 577–582. 17300962 10.1016/j.neuroimage.2006.12.033

[bibr2-0269881117736915] Anderson CA Deuser WE DeNeve KM (1995) Hot temperatures, hostile affect, hostile cognition, and arousal: Tests of a general model of affective aggression. Pers Soc Psychol Bull 21: 434–448.

[bibr3-0269881117736915] Bajo R Pusil S Lopez ME et al (2015) Scopolamine effects on functional brain connectivity: A pharmacological model of Alzheimer’s disease. Sci Rep 5: 9748. 26130273 10.1038/srep09748 PMC4486953

[bibr4-0269881117736915] Bastiaansen MC Knosche TR (2000) Tangential derivative mapping of axial MEG applied to event-related desynchronization research. Clin Neurophysiol 111: 1300–1305. 10880806 10.1016/s1388-2457(00)00272-8

[bibr5-0269881117736915] Brookes MJ Hale JR Zumer JM et al (2011) Measuring functional connectivity using MEG: Methodology and comparison with fcMRI. Neuroimage 56: 1082–1104. 21352925 10.1016/j.neuroimage.2011.02.054 PMC3224862

[bibr6-0269881117736915] Buckner RL Sepulcre J Talukdar T et al (2009) Cortical hubs revealed by intrinsic functional connectivity: Mapping, assessment of stability, and relation to Alzheimer’s disease. J Neurosci 29: 1860–1873. 19211893 10.1523/JNEUROSCI.5062-08.2009 PMC2750039

[bibr7-0269881117736915] Colclough GL Brookes MJ Smith SM et al (2015) A symmetric multivariate leakage correction for MEG connectomes. Neuroimage 117: 439–448. 25862259 10.1016/j.neuroimage.2015.03.071 PMC4528074

[bibr8-0269881117736915] Creutzfeldt OD Arnold PM Becker D et al (1976) EEG changes during spontaneous and controlled menstrual cycles and their correlation with psychological performance. Electroencephalogr Clin Neurophysiol 40: 113–131. 55353 10.1016/0013-4694(76)90157-7

[bibr9-0269881117736915] Dingledine R Borges K Bowie D et al (1999) The glutamate receptor ion channels. Pharmacol Rev 51: 7–62. 10049997

[bibr10-0269881117736915] Downey D Dutta A McKie S et al (2016) Comparing the actions of lanicemine and ketamine in depression: Key role of the anterior cingulate. Eur Neuropsychopharmacol 26: 994–1003. 27133029 10.1016/j.euroneuro.2016.03.006

[bibr11-0269881117736915] FDA (2012) Fycompa Full Prescribing Information. Available at: http://www.accessdata.fda.gov/drugsatfda_docs/label/2012/202834lbl.pdf (accessed 16 June 2015).

[bibr12-0269881117736915] Franzen JD Heinrichs-Graham E White ML et al (2013) Atypical coupling between posterior regions of the default mode network in attention-deficit/hyperactivity disorder: A pharmaco-magnetoencephalography study. J Psychiatry Neurosci 38: 333–340. 23611175 10.1503/jpn.120054 PMC3756117

[bibr13-0269881117736915] Goff DC Coyle JT (2014) The emerging role of glutamate in the pathophysiology and treatment of schizophrenia. Am J Psychiatry 158: 1367–1377. 10.1176/appi.ajp.158.9.1367 11532718

[bibr14-0269881117736915] Gsell W Burke M Wiedermann D et al (2006) Differential effects of NMDA and AMPA glutamate receptors on functional magnetic resonance imaging signals and evoked neuronal activity during forepaw stimulation of the rat. J Neurosci 26: 8409–8416. 16914666 10.1523/JNEUROSCI.4615-05.2006 PMC6674350

[bibr15-0269881117736915] Hall EL Woolrich MW Thomaz CE et al (2013) Using variance information in magnetoencephalography measures of functional connectivity. Neuroimage 67: 203–212. 23165323 10.1016/j.neuroimage.2012.11.011

[bibr16-0269881117736915] Hall SD Barnes GR Furlong PL et al (2010) Neuronal network pharmacodynamics of GABAergic modulation in the human cortex determined using pharmaco-magnetoencephalography. Hum Brain Mapp 31: 581–594. 19937723 10.1002/hbm.20889 PMC3179593

[bibr17-0269881117736915] Hall SD Stanford IM Yamawaki N et al (2011) The role of GABAergic modulation in motor function related neuronal network activity. Neuroimage 56: 1506–1510. 21320607 10.1016/j.neuroimage.2011.02.025

[bibr18-0269881117736915] Hamandi K Myers J Muthukumaraswamy S (2014) Tiagabine-induced stupor - more evidence for an encephalopathy. Epilepsy Behav 31: 196–197. 24440890 10.1016/j.yebeh.2013.12.027

[bibr19-0269881117736915] Heinrichs-Graham E Kurz MJ Becker KM et al (2014) Hypersynchrony despite pathologically reduced beta oscillations in patients with Parkinson’s disease: A pharmaco-magnetoencephalography study. J Neurophysiol 112: 1739–1747. 25008416 10.1152/jn.00383.2014 PMC4157173

[bibr20-0269881117736915] Herrero JL Gieselmann MA Sanayei M et al (2013) Attention-induced variance and noise correlation reduction in macaque V1 is mediated by NMDA receptors. Neuron 78: 729–739. 23719166 10.1016/j.neuron.2013.03.029 PMC3748348

[bibr21-0269881117736915] Hillebrand A Barnes GR Bosboom JL et al (2012) Frequency-dependent functional connectivity within resting-state networks: An atlas-based MEG beamformer solution. Neuroimage 59: 3909–3921. 22122866 10.1016/j.neuroimage.2011.11.005 PMC3382730

[bibr22-0269881117736915] Hong LE Summerfelt A Buchanan RW et al (2009) Gamma and delta neural oscillations and association with clinical symptoms under subanesthetic ketamine. Neuropsychopharmacology 35: 632–640. 19890262 10.1038/npp.2009.168 PMC3055615

[bibr23-0269881117736915] Hynd MR Scott HL Dodd PR (2004) Glutamate-mediated excitotoxicity and neurodegeneration in Alzheimer’s disease. Neurochem Int 45: 583–595. 15234100 10.1016/j.neuint.2004.03.007

[bibr24-0269881117736915] Korostenskaja M Nikulin VV Kicic D et al (2007) Effects of NMDA receptor antagonist memantine on mismatch negativity. Brain Res Bull 72: 275–283. 17452287 10.1016/j.brainresbull.2007.01.007

[bibr25-0269881117736915] Lazarewicz MT Ehrlichman RS Maxwell CR et al (2009) Ketamine modulates theta and gamma oscillations. J Cogn Neurosci 22: 1452–1464. 10.1162/jocn.2009.21305 19583475

[bibr26-0269881117736915] Liao W Zhang Z Pan Z et al (2010) Altered functional connectivity and small-world in mesial temporal lobe epilepsy. PLoS One 5: e8525. 20072616 10.1371/journal.pone.0008525 PMC2799523

[bibr27-0269881117736915] Licata SC Lowen SB Trksak GH et al (2011) Zolpidem reduces the blood oxygen level-dependent signal during visual system stimulation. Prog Neuropsychopharmacol Biol Psychiatry 35: 1645–1652. 21640782 10.1016/j.pnpbp.2011.05.015 PMC3154455

[bibr28-0269881117736915] Linssen AM Sambeth A Vuurman EF et al (2014) Cognitive effects of methylphenidate and levodopa in healthy volunteers. Eur Neuropsychopharmacol 24: 200–206. 24119823 10.1016/j.euroneuro.2013.09.009

[bibr29-0269881117736915] Lopes de Silva FH (2010) Electrophysiological basis of MEG signals. In: Hansen P Kringelbach M Salmelin R (eds) MEG: An Introduction to Methods. New York, NY: Oxford University Press.

[bibr30-0269881117736915] Lozano-Soldevilla D ter Huurne N Cools R et al (2014) GABAergic modulation of visual gamma and alpha oscillations and its consequences for working memory performance. Curr Biol 24: 2878–2887. 25454585 10.1016/j.cub.2014.10.017

[bibr31-0269881117736915] Luo C Qiu C Guo Z et al (2012) Disrupted functional brain connectivity in partial epilepsy: A resting-state fMRI study. PLoS One 7: e28196. 10.1371/journal.pone.0028196 PMC3252302 22242146

[bibr32-0269881117736915] Magazzini L Muthukumaraswamy SD Campbell AE et al (2016) Significant reductions in human visual gamma frequency by the gaba reuptake inhibitor tiagabine revealed by robust peak frequency estimation. Hum Brain Mapp 37: 3882–3896. 27273695 10.1002/hbm.23283 PMC5082569

[bibr33-0269881117736915] Martin CS Earleywine M Musty RE et al (1993) Development and validation of the biphasic alcohol effects scale. Alcohol Clin Exp Res 17: 140–146. 8452195 10.1111/j.1530-0277.1993.tb00739.x

[bibr34-0269881117736915] McEntee WJ Crook TH (1993) Glutamate: Its role in learning, memory, and the aging brain. Psychopharmacology 111: 391–401. 7870979 10.1007/BF02253527

[bibr35-0269881117736915] Meldrum BS (1996) Update on the mechanism of action of antiepileptic drugs. Epilepsia 37: S4–S11. 10.1111/j.1528-1157.1996.tb06038.x 8941036

[bibr36-0269881117736915] Moran RJ Symmonds M Stephan KE et al (2011) An in vivo assay of synaptic function mediating human cognition. Curr Biol 21: 1320–1325. 21802302 10.1016/j.cub.2011.06.053 PMC3153654

[bibr37-0269881117736915] Muthukumaraswamy SD (2014) The use of magnetoencephalography in the study of psychopharmacology (pharmaco-MEG). J Psychopharmacol 28: 815–829. 24920134 10.1177/0269881114536790

[bibr38-0269881117736915] Muthukumaraswamy SD Carhart-Harris RL Moran RJ et al (2013) Broadband cortical desynchronization underlies the human psychedelic state. J Neurosci 33: 15171–15183. 24048847 10.1523/JNEUROSCI.2063-13.2013 PMC6618409

[bibr39-0269881117736915] Muthukumaraswamy SD Routley B Droog W et al (2016) The effects of AMPA blockade on the spectral profile of human early visual cortex recordings studied with non-invasive MEG. Cortex 81: 266–275. 27209006 10.1016/j.cortex.2016.03.004

[bibr40-0269881117736915] Nichols TE Holmes AP (2002) Nonparametric permutation tests for functional neuroimaging: A primer with examples. Hum Brain Mapp 15: 1–25. 11747097 10.1002/hbm.1058 PMC6871862

[bibr41-0269881117736915] Northoff G Richter A Bermpohl F et al (2005) NMDA hypofunction in the posterior cingulate as a model for schizophrenia: An exploratory ketamine administration study in fMRI. Schizophr Res 72: 235–248. 15560968 10.1016/j.schres.2004.04.009

[bibr42-0269881117736915] Nutt D Wilson S Lingford-Hughes A et al (2015) Differences between magnetoencephalographic (MEG) spectral profiles of drugs acting on GABA at synaptic and extrasynaptic sites: A study in healthy volunteers. Neuropharmacology 88: 155–163. 25195191 10.1016/j.neuropharm.2014.08.017

[bibr43-0269881117736915] Oke OO Magony A Anver H et al (2010) High-frequency gamma oscillations coexist with low-frequency gamma oscillations in the rat visual cortex in vitro. Eur J Neurosci 31: 1435–1445. 20384769 10.1111/j.1460-9568.2010.07171.x

[bibr44-0269881117736915] Olney JW Farber NB (1995) Glutamate receptor dysfunction and schizophrenia. Arch Gen Psychiatry 52: 998–1007. 7492260 10.1001/archpsyc.1995.03950240016004

[bibr45-0269881117736915] Oostenveld R Fries P Maris E et al (2011) FieldTrip: Open source software for advanced analysis of MEG, EEG, and invasive electrophysiological data. Comput Intell Neurosci 2011: 156869. 21253357 10.1155/2011/156869 PMC3021840

[bibr46-0269881117736915] Riedel G Platt B Micheau J (2003) Glutamate receptor function in learning and memory. Behav Brain Res 140: 1–47. 12644276 10.1016/s0166-4328(02)00272-3

[bibr47-0269881117736915] Rogawski MA Hanada T (2013) Preclinical pharmacology of perampanel, a selective non-competitive AMPA receptor antagonist. Acta Neurol Scand Suppl 197: 19–24. 10.1111/ane.12100 PMC4506647 23480152

[bibr48-0269881117736915] Sanacora G Smith MA Pathak S et al (2014) Lanicemine: A low-trapping NMDA channel blocker produces sustained antidepressant efficacy with minimal psychotomimetic adverse effects. Mol Psychiatry 19: 978–985. 24126931 10.1038/mp.2013.130 PMC4195977

[bibr49-0269881117736915] Sanacora G Treccani G Popoli M (2012) Towards a glutamate hypothesis of depression: An emerging frontier of neuropsychopharmacology for mood disorders. Neuropharmacology 62: 63–77. 21827775 10.1016/j.neuropharm.2011.07.036 PMC3205453

[bibr50-0269881117736915] Saunders JB Aasland OG Babor TF et al (1993) Development of the alcohol use disorders identification test (AUDIT): WHO collaborative project on early detection of persons with harmful alcohol consumption-II. Addiction 88: 791–804. 8329970 10.1111/j.1360-0443.1993.tb02093.x

[bibr51-0269881117736915] Saxena N Muthukumaraswamy SD Diukova A et al (2013) Enhanced stimulus-induced gamma activity in humans during propofol-induced sedation. PLoS One 8: e57685. 23483920 10.1371/journal.pone.0057685 PMC3590225

[bibr52-0269881117736915] Schuckit MA (1980) Self-rating of alcohol intoxication by young men with and without family histories of alcoholism. J Stud Alcohol 41: 242–249. 7374142 10.15288/jsa.1980.41.242

[bibr53-0269881117736915] Sebban C Tesolin-Decros B Ciprian-Ollivier J et al (2002) Effects of phencyclidine (PCP) and MK 801 on the EEGq in the prefrontal cortex of conscious rats; antagonism by clozapine, and antagonists of AMPA-, α1- and 5-HT2A-receptors. Br J Pharmacol 135: 65–78. 11786481 10.1038/sj.bjp.0704451 PMC1573114

[bibr54-0269881117736915] Shaw AD Saxena N Jackson L et al (2015) Ketamine amplifies induced gamma frequency oscillations in the human cerebral cortex. Eur Neuropsychopharmacol 25: 1136–1146. 26123243 10.1016/j.euroneuro.2015.04.012

[bibr55-0269881117736915] Solis-Ortiz S Ramos J Arce C et al (1994) EEG oscillations during menstrual cycle. Int J Neurosci 76: 279–292. 7960484 10.3109/00207459408986010

[bibr56-0269881117736915] Templeton D (2010) Pharmacokinetics of Perampanel, a Highly Selective AMPA-Type Glutamate Receptor Antagonist Following Once- and Multiple- Daily Dosing. Presented at the 9th European Congress on Epileptology, Rhodes, Greece, 28 June 2010.

[bibr57-0269881117736915] Tomasi D Volkow ND (2011) Association between functional connectivity hubs and brain networks. Cereb Cortex 21: 2003–2013. 21282318 10.1093/cercor/bhq268 PMC3165965

[bibr58-0269881117736915] Traynelis SF Wollmuth LP McBain CJ et al (2010) Glutamate receptor ion channels: Structure, regulation, and function. Pharmacol Rev 62: 405–496. 20716669 10.1124/pr.109.002451 PMC2964903

[bibr59-0269881117736915] Wilson TW Franzen JD Heinrichs-Graham E et al (2013) Broadband neurophysiological abnormalities in the medial prefrontal region of the default-mode network in adults with ADHD. Hum Brain Mapp 34: 566–574. 22102400 10.1002/hbm.21459 PMC6870131

[bibr60-0269881117736915] Zaccara G Giovannelli F Cincotta M et al (2013) The adverse event profile of perampanel: Meta-analysis of randomized controlled trials. Eur J Neurol 20: 1204–1211. 23607817 10.1111/ene.12170

