# Supplementary material for: The effects of AMPA receptor blockade on resting magnetoencephalography recordings
Source: J Psychopharmacol. 2017 Oct 31;31(12):1527–36. doi: 10.1177/0269881117736915 (PMC5987991; doi:10.1177/0269881117736915)
Supplement: Supplementary material [file JOP736915_Supplementary_Material.pdf]

Supplementary materials

Supplementary table 1 - Descriptive statistics for psychometric questionnaires.

SHAS		Pre-Dose	Post-Dose
Perampanel	Mean	43.2	195.5
	SD	46.5	159.1
Placebo	Mean	48.5	45.2
	SD	77.9	73.8
BAES - Sedative			
Perampanel	Mean	12.0	25.5
	SD	5.4	11.5
Placebo	Mean	11.3	11.9
	SD	4.9	7.3
BAES - Stimulant			
Perampanel	Mean	30.7	23.8
	SD	14.4	12.1
Placebo	Mean	31.4	27.3
	SD	11.7	14.0
SHS			
Perampanel	Mean	55.3	58.0
	SD	9.2	12.7
Placebo	Mean	54.2	53.4
	SD	9.2	9.5

Supplementary table 2 - Biphasic Alcohol Effects Scale (BAES) item averages

BAES - Sedative subscale				
	Perampanel		Placebo	
	Pre-dose	Post-dose	Pre-dose	Post-dose
Difficulty in concentrating	1.95	4.05	1.5	1.7
Down	1.6	1.9	1.55	1.45
Heavy head	1.45	3.75	1.6	1.65
Inactive	2.45	3.9	2.1	1.9
Sedated	1.35	4.1	1.4	1.5
Slow thoughts	1.4	3.7	1.45	1.75
Sluggish	1.8	4.1	1.65	1.9
Total	12	25.5	11.25	11.85

BAES - Stimulant subscale				
	Perampanel		Placebo	
	Pre-dose	Post-dose	Pre-dose	Post-dose
Elated	3.55	3.5	4.2	3.55
Energized	4.35	2.8	4.75	3.85
Excited	4.55	3.1	3.95	3.65
Stimulated	4.2	3.25	4.6	3.9
Talkative	4.9	4.2	4.65	4.45
Up	5.1	4.25	5.5	4.65
Vigorous	4	2.7	3.7	3.25
Total	30.65	23.8	31.35	27.3

Supplementary table 3 - Subjective High Assessment Scale (SHAS) item averages

SHAS

	Perampanel		Placebo	
	Pre-dose	Post-dose	Pre-dose	Post-dose
Uncomfortable	15.7	6.1	8.8	5.5
High	5.1	8.2	7.2	7.3
Clumsy	5.1	21.1	5.8	3.4
Muddled or confused	3.8	12.4	3.1	2.9
Slurred speech	0.7	7.6	1.0	1.6
Dizzy	0.0	18.9	0.9	2.5
Nauseated	0.3	4.5	2.0	1.7
Drunk or intoxicated	0.0	15.8	0.4	0.6
Sleepy	9.7	40.8	14.4	13.5
Distorted sense of time	0.0	7.3	0.9	3.8
Effects of alcohol	0.0	13.7	0.4	0.0
Difficulty concentrating	2.7	27.4	3.1	1.6
Feeling of floating	0.3	12.1	0.9	1.2

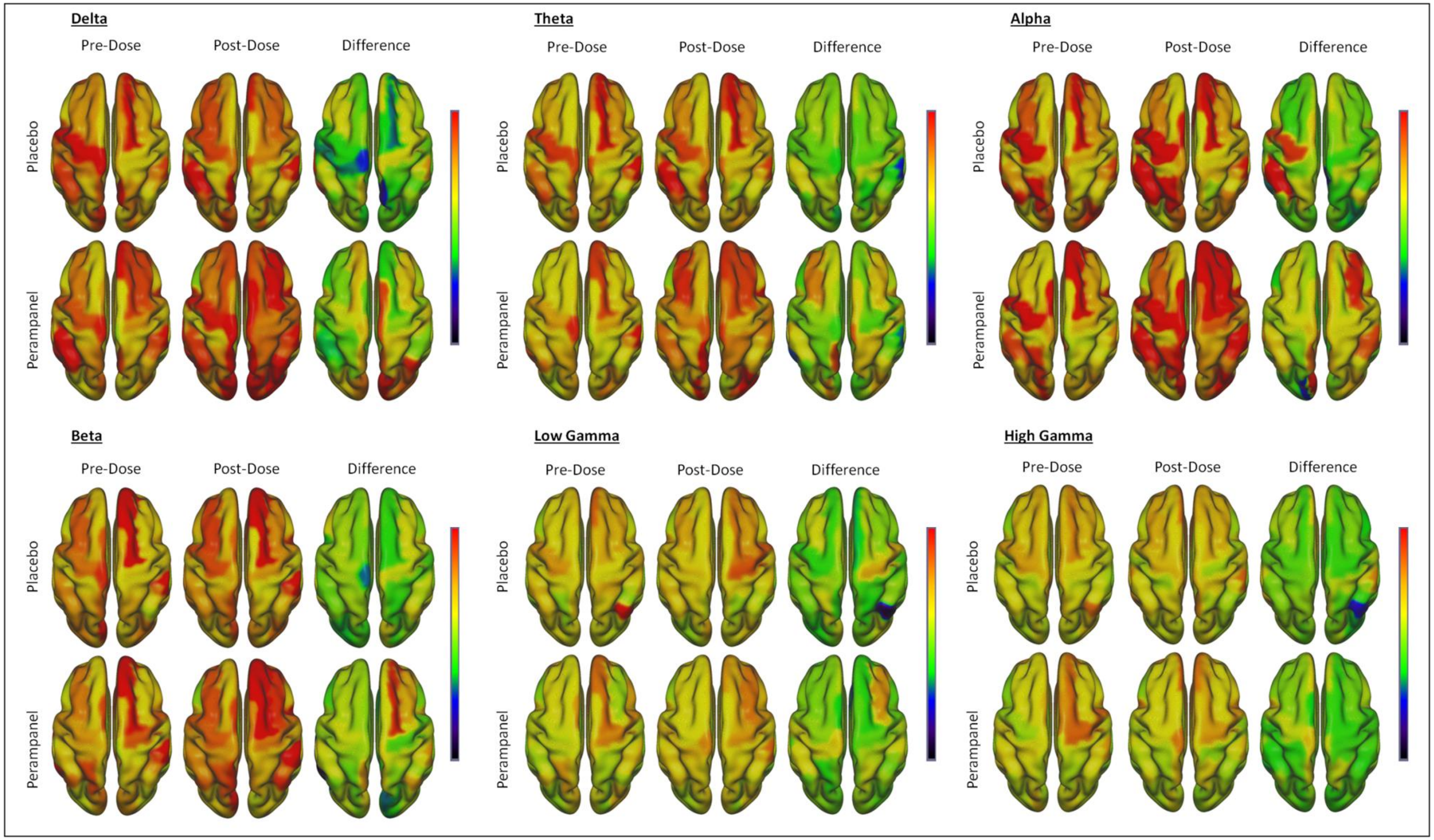

Supplementary Figure 1 – Source power maps. For each frequency band, power in each of the 90 AAL atlas regions is plotted on a template brain for pre-dose and post-dose timepoints for both placebo and perampanel, along with the post-dose – pre-dose power differences. Units are arbitrary due to the beamformer weights normalisation process that is needed to correct for biases introduced by non-uniform sensor-noise projection
